# Angiotensin-(1-7) Prevents Lipopolysaccharide-Induced Autophagy via the Mas Receptor in Skeletal Muscle

**DOI:** 10.3390/ijms21249344

**Published:** 2020-12-08

**Authors:** Juan Carlos Rivera, Johanna Abrigo, Franco Tacchi, Felipe Simon, Enrique Brandan, Robson A. Santos, Michael Bader, Mario Chiong, Claudio Cabello-Verrugio

**Affiliations:** 1 Laboratory of Muscle Pathology, Fragility and Aging, Department of Biological Sciences, Faculty of Life Sciences, Universidad Andres Bello, Santiago 8370146, Chile; jcfriverac@gmail.com (J.C.R.); j.abrigo.leon@gmail.com (J.A.); f.tacchifernandez@uandresbello.edu (F.T.); 2 Millennium Institute on Immunology and Immunotherapy, Santiago 8370146, Chile; fsimon@unab.cl; 3 Center for the Development of Nanoscience and Nanotechnology (CEDENNA), Universidad de Santiago de Chile, Santiago 8350709, Chile; 4 Laboratory of Integrative Physiopathology, Department of Biological Sciences, Faculty of Life Sciences, Universidad Andres Bello, Santiago 8370186, Chile; 5 Millennium Nucleus of Ion Channel-Associated Diseases (MiNICAD), Universidad de Chile, Santiago 8380453, Chile; 6 Center for Cell Regulation and Pathology (CRCP), Center for Regeneration and Aging (CARE), Laboratory of Cell Differentiation and Pathology, Department of Cell and Molecular Biology, Faculty of Biological Sciences, P. Universidad Católica de Chile, Santiago 8330077, Chile; ebrandan@bio.puc.cl; 7 Fundación Ciencia & Vida, Santiago 7780272, Chile; 8 National Institute in Science and Technology in Nanobiopharmaceutics, Department of Physiology and Biophysics, Federal University of Minas Gerais (UFMG), Belo Horizonte 31270-901, Brazil; robsonsant@gmail.com; 9 Max-Delbrück-Center for Molecular Medicine, 13125 Berlin, Germany; mbader@mdc-berlin.de; 10 Institute for Biology, University of Lübeck, 23562 Lübeck, Germany; 11 Charité University Medicine, 10117 Berlin, Germany; 12 German Center for Cardiovascular Research (DZHK), 10785 Berlin, Germany; 13 Advanced Center for Chronic DiSeases (ACCDiS), Universidad de Chile, Santiago 8380492, Chile; mchiong@ciq.uchile.cl

**Keywords:** renin-angiotensin system, muscle wasting, autophagy, LPS

## Abstract

Skeletal muscle atrophy, which occurs in lipopolysaccharide (LPS)-induced sepsis, causes a severe muscle function reduction. The increased autophagy contributes to sepsis-induced skeletal muscle atrophy in a model of LPS injection, increasing LC3II/LC3I ratio, autophagy flux, and autophagosomes. Angiotensin-(1-7) (Ang-(1-7)) has anti-atrophic effects via the Mas receptor in skeletal muscle. However, the impact of Ang-(1-7) on LPS-induced autophagy is unknown. In this study, we determined the effect of Ang-(1-7) on sepsis-induced muscle autophagy. C57BL6 wild-type (WT) mice and mice lacking the Mas receptor (KO Mas) were injected with LPS together with the systemic administration of Ang-(1-7) to determine autophagy in skeletal muscle. We also evaluated autophagy and p38 and c-Jun N-terminal kinase (JNK)activation. Our results show that Ang-(1-7) prevents LPS-induced autophagy in the diaphragm, tibialis anterior, and gastrocnemius of WT mice, which is demonstrated by a decrease in the LC3II/LC3I ratio and mRNA levels of *lc3b* and *ctsl*. This effect was lost in KO Mas mice, suggesting the role of the Mas receptor. The results in C2C12 cells show that Ang-(1-7) reduces several LPS-dependent effects, such as autophagy (LC3II/LC3I ratio, autophagic flux, and autophagosomes), activation of p38 and JNK, B-cell lymphoma-2 (BCL2) phosphorylation, and disassembly of the Beclin1/BCL2 complex. In conclusion, Ang-(1-7)/Mas receptor reduces LPS-induced autophagy in skeletal muscle. In vitro assays indicate that Ang-(1-7) prevents LPS-induced autophagy and modifies the MAPK signaling and the disassembly of a complex involved at the beginning of autophagy.

## 1. Introduction

Skeletal muscle atrophy is the progressive loss of skeletal muscle mass and strength [1,2,3,4]. Different stimuli are responsible for skeletal muscle atrophy. One is sepsis induced by endotoxin lipopolysaccharide (LPS), a cell wall component of gram-negative bacteria [5]. LPS triggers an exacerbated inflammatory response that results in severe muscle wasting [6,7,8,9]. Patients in intensive care units are one of the most affected groups by sepsis-induced muscle wasting. Sepsis produces damage in the muscles necessary for locomotion, such as the tibialis anterior (TA) and gastrocnemius (GA). However, the most affected muscle is the diaphragm (DFG), which results in the need for a mechanical ventilator to assist in respiration [10].

In LPS-induced skeletal muscle atrophy, protein degradation increases through the ubiquitin-proteasome system (UPS), and autophagy increases through toll-like receptor 4 signaling [11,12]. Autophagy is a homeostatic mechanism via lysosomal machinery for the degradation and recycling of the bulk cytoplasm of long-lived proteins and organelles incorporated into double-membrane vesicles called autophagosomes [3,13]. An essential protein in autophagy is microtubule-associated proteins 1A/1B light chain 3B (LC3B) [14]. Post-translational modifications of LC3B protein are required for biogenesis, elongation, and maturation of the autophagosome. LC3B is cleaved and then conjugated to phosphatidylethanolamine (PE) to generate LC3-PE (a membrane-bound form of LC3, also referred to as LC3II). The level of LC3II is known to be correlated with the number of autophagosomes [14]. During the post-translational LC3B processing, two forms are produced: LC3I (inactive protein) and LC3II (active form). The LC3II/LC3I ratio allows analyzing the conversion to an active form of LC3B (LC3I to LC3II) as autophagy activation [14]. The guidelines for monitoring autophagy [15] establish that LC3B protein processing is the mostly marker to study autophagy and autophagosome [16]. Besides, several signaling pathways have been described to initiate and regulate autophagy, and amongst them are the mitogen-activated protein kinases (MAPKs), specifically p38 and JNK. These kinases are crucial to activating autophagy in food deprivation [17,18]. Regarding the intracellular mechanism regulatory of autophagy, MAPKs are involved in the disassembly of the Beclin1/BCL-2 complex via specific phosphorylation of Beclin1 and BCL-2 to enable the interaction of Beclin1 with the PI3K class III Vps34 complex [19]. Interestingly, LPS also increases p38 and JNK phosphorylation in skeletal muscle cells [9,11,20].

The renin-angiotensin system (RAS) is mainly composed of classical and non-classical axes, which have been described as regulators of skeletal muscle mass [21]. On the one hand, the classical RAS through the principal peptide effector Angiotensin II (Ang II) and the AT1 receptor produces skeletal muscle atrophy [22]. On the other hand, in the non-classical RAS axis, the G-protein-coupled Mas receptor and its ligand angiotensin (1-7) (Ang-(1-7)) are the principal effectors [22,23]. There is increased Mas expression in skeletal muscle atrophy caused by immobilization, sarcopenia, and sepsis [22,24]. Interestingly, Ang-(1-7) possesses anti-atrophic effects via Mas receptor activation [24,25]. We have previously demonstrated that Ang-(1-7) has anti-atrophic properties via the Mas receptor by restoring parameters, such as muscle strength and the cross-sectional area of muscle fiber, and reducing the E3 ubiquitin ligases muscle RING-finger protein-1 (MuRF-1) and atrogin-1/MAFbx (atrogin-1) [9]. Additionally, Ang-(1-7) reduces p38 phosphorylation via Mas receptor activation [9].

In this study, we evaluated the role of Ang-(1-7) and the Mas receptor in LPS-induced autophagy in skeletal muscle and Ang-(1-7)/Mas receptor effect on MAPK phosphorylation and the disassembly of the Beclin1/BCL-2 complex in skeletal muscle cells.

## 2. Results

### 2.1. Angiotensin-(1-7) Prevented the Decline of Muscle Function and Tetanic Force via the Mas Receptor in LPS-Treated Mice

**Figure 1:**
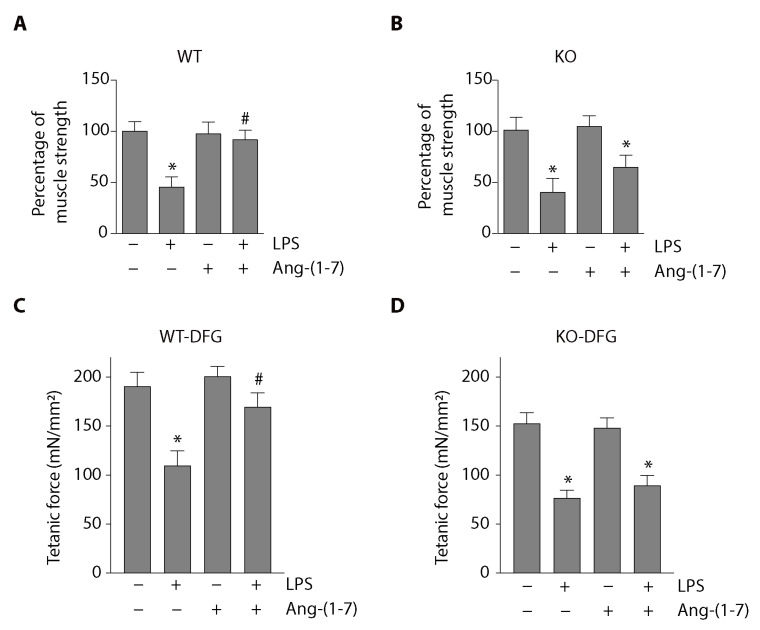
Angiotensin-(1-7) (Ang-(1-7)) prevented a lipopolysaccharide (LPS)-induced reduction in muscle function and tetanic force via the Mas receptor in mice. Wild-type (WT) and Mas knockout (Mas KO) C57BL/6J male mice were treated with the vehicle, LPS, Ang-(1-7), or LPS + Ang-(1-7) for 14 days. Then, limb muscle strength was measured using the weightlifting test in WT (**A**) and Mas KO (**B**) mice. The score was normalized by body weight, and the results were represented in the graph as mean ± S.E. Nine animals per group were used in these experiments (*, *p* < 0.05 vs. vehicle. #, *p* < 0.05 vs. LPS). The tetanic force was measured in isolated diaphragm (DFG) from WT (**C**) and Mas KO (**D**) mice after 24 h of treatments with LPS and/or Ang-(1-7). The values are expressed as mean ± S.E. Four animals per group were used in these experiments (*, *p* < 0.05 vs. vehicle. #, *p* < 0.05 vs. LPS). Plus (+) and minus (−) symbols indicated in the figures or graphics mean presence or absence of treatment.

We analyzed the effect of Ang-(1-7) and the Mas receptor on muscle function in a mouse model of LPS-induced muscle wasting. The weightlifting test results shown in Figure 1A indicate that wild-type (WT) mice injected with LPS presented a 55% reduction in muscle function compared with the vehicle group’s WT mice. The same figure shows that this decrease was prevented by Ang-(1-7) administration.

To evaluate the Mas receptor’s role in the prevention of LPS-induced muscle function decline observed in Ang-(1-7) administration, we used mice deficient in Mas expression (Mas KO mice). In these mice, LPS also reduced muscle strength (60% in the vehicle group). However, Ang-(1-7) was unable to recover the muscle function to the normal levels (Figure 1B) in contrast with the effect observed in WT mice.

We also evaluated the tetanic force in isolated diaphragm (DFG) muscle from LPS-treated mice for 24 h. Figure 1C shows that the LPS-induced decrease in strength (control: 191 ± 14; LPS: 110 ± 15; Ang-(1-7): 201 ± 10 mN/mm^2^) was partially prevented when mice were treated with Ang-(1-7) (LPS + Ang-(1-7): 170 ± 14 mN/mm^2^). In Figure 1D, we observed that the protective effect of Ang-(1-7) on the tetanic force was abolished when the experiments were performed in Mas KO mice (control: 152 ± 11; LPS: 76 ± 8; Ang-(1-7): 148 ± 10; LPS + Ang-(1-7): 89 ± 10 mN/mm^2^).

These results confirm that Ang-(1-7) prevented LPS-induced loss of muscle function and strength via the Mas receptor. In addition, these results obtained in Mas KO mice are identical and consistent with findings previously reported using WT mice treated with the antagonist of Mas receptor A779 in which muscle atrophy and wasting were abolished to be recovered by Ang-(1-7) [9].

### 2.2. Angiotensin-(1-7) Decreased LPS-Induced Autophagy in the Diaphragm, Tibialis Anterior, and Gastrocnemius Muscles through a Mas Receptor-Dependent Mechanism

**Figure 2:**
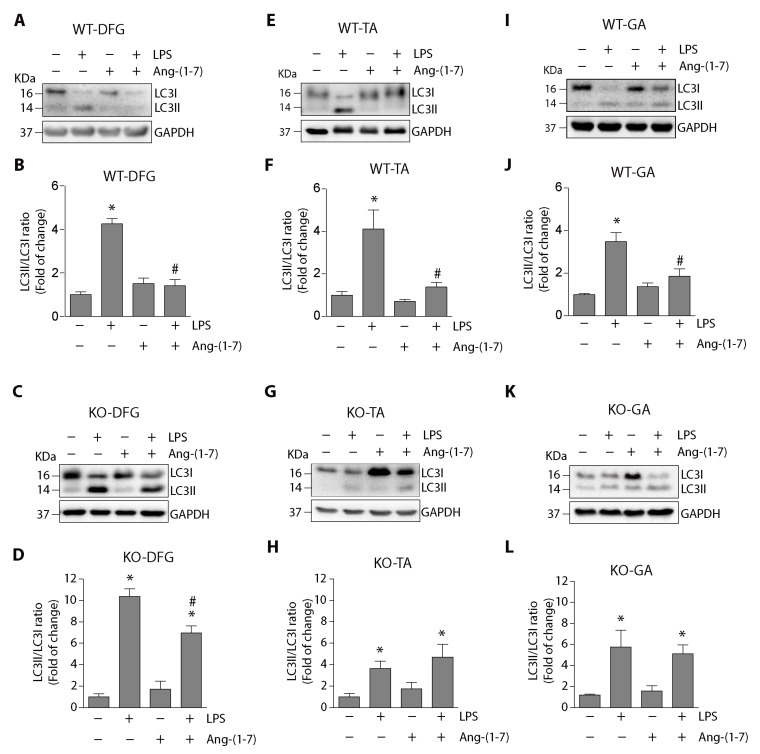
Ang-(1-7) decreased the LPS-induced LC3II/LC3I ratio via the Mas receptor in muscles from mice. C57BL/6J wild-type or Mas KO male mice were treated with the vehicle, LPS, Ang-(1-7), or LPS + Ang-(1-7) for 18 h. At the end of the experiment, the mice were sacrificed, and the muscle was excised and homogenized to evaluate LC3I and LC3II protein levels through Western blot analysis in DFG (**A**,**C**), TA (**E**,**G**), and GA (**I**,**K**). Glyceraldehyde-3-phosphate dehydrogenase (GAPDH) levels are shown as loading control. Molecular weight markers are depicted in kDa. The quantitative analysis of the experiments is shown for DFG (**B**,**D**), TA (**F**,**H**), and GA (**J**,**L**). The results were represented as LC3II/LC3I ratio and expressed as the mean ± S.E. (the fold of change relative to the vehicle group). Nine animals per group were used in these experiments (*, *p* < 0.05 vs. vehicle. #, *p* < 0.05 vs. LPS). Plus (+) and minus (−) symbols indicated in the figures or graphics mean presence or absence of treatment.

We evaluated the effect of Ang-(1-7) on LPS-induced autophagy in mice. The protein levels of LC3II and LC3I were detected using Western blot in muscles from WT mice. Figure 2A,E, I show that the LPS-induced increase in LC3II/LC3I ratio in DFG, tibialis anterior (TA) and gastrocnemius (GA) muscles were diminished by Ang-(1-7) administration. A quantitative analysis of the LC3II/LC3I ratio is shown in Figure 2B,F,J, which indicate that Ang-(1-7) prevented the increase induced by LPS from 4.2 ± 0.2-fold to 1.4 ± 0.2-fold in DFG (Figure 2B), from 4.1 ± 0.9-fold to 1.3 ± 0.2-fold in TA (Figure 2F) and from 3.4 ± 0.4-fold to 1.8 ± 0.3-fold in GA (Figure 2J).

To evaluate the Mas receptor’s role, Mas KO mice were treated with LPS in the absence or presence of Ang-(1-7). Figure 2C shows that the LPS-dependent increase (relative to its control) in the LC3II/LC3I ratio was decreased to a minor degree with Ang-(1-7) treatment (10.3 ± 0.7 to 6.9 ± 0.6-fold) compared with the case in WT mice in DFG (Figure 2D). The results in TA (Figure 2G) and GA (Figure 2K) muscles from LPS-treated Mas KO mice show that Ang-(1-7) did not affect the LPS-induced increase in the LC3II/LC3I ratio. Quantitative analysis of these data in TA muscle indicates that the LPS-induced increase in the LC3II/LC3I ratio (3.8 ± 0.6-fold) was maintained despite Ang-(1-7) administration (4.8 ± 1.6-fold) (Figure 2H). In the GA muscles, the LPS-induced increase in the LC3II/LC3I ratio (5.7 ± 1.5-fold) was not altered with Ang-(1-7) treatment (5.1 ± 0.8-fold) (Figure 2L).

As autophagy involves cargo proteins’ participation, we evaluated the effect of LPS and Ang-(1-7) treatments on p62/SQSTM1 protein levels. The results indicate that p62/SQSTM1 levels are unchanged for LPS or Ang-(1-7) treatments in DFG (Figure S1A–C), TA (Figure S1D–F), and GA (Figure S1G–I) from WT and Mas KO mice.

We also evaluated the Ang-(1-7) effect on the LPS-modulated gene expression of *Lc3b* and *Ctsl*, two genes upregulated by autophagy activation. In WT mice, *lc3b* is increased to 2.0 ± 0.2-fold by LPS, whereas it is decreased to 1.0 ± 0.1-fold by Ang-(1-7) administration (Figure S2A). Similar results were obtained for *ctsl* expression in WT mice: Ang-(1-7) decreased the LPS-induced mRNA levels from 5.1 ± 0.8-fold to 2.8 ± 0.6-fold (Figure S2B). In the KO Mas mice, the effect of Ang-(1-7) was lost for *lc3b* expression [LPS: 2.6 ± 0.5-fold vs. LPS + Ang-(1-7): 2.1 ± 0.1-fold] (Figure S2C) and *ctsl* expression [LPS: 4.4 ± 0.6-fold vs. LPS + Ang-(1-7): 4.4 ± 0.6-fold] (Figure S2D).

These results demonstrate that Ang-(1-7) decreased LPS-induced autophagy via the skeletal muscle’s Mas receptor.

### 2.3. Angiotensin-(1-7) Decreased LPS-Induced Autophagy in Skeletal Muscle Cells

**Figure 3:**
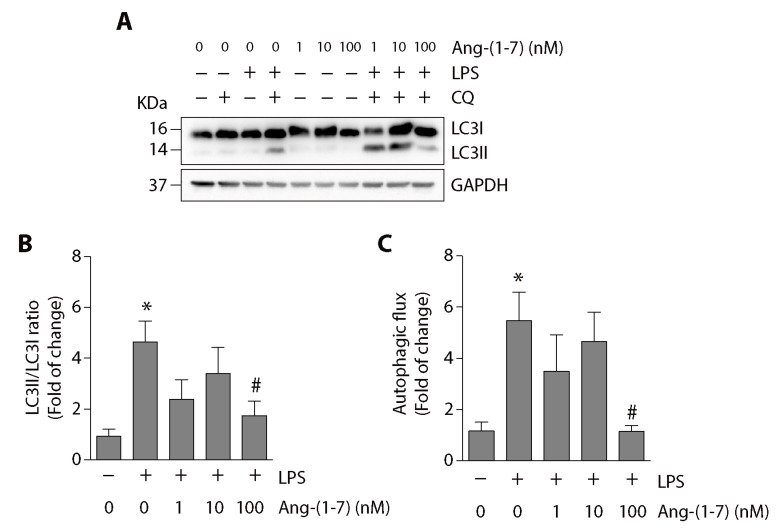
Ang-(1-7) reduced the LPS-induced LC3II/LC3I ratio and autophagic flux in C2C12 myotubes. C2C12 cells were pre-treated with or without Ang-(1-7) (1, 10, and 100 nM) for 30 min and then incubated in the absence or presence of chloroquine (CQ; 50 μM) for 5 min. Next, the cells were treated with the vehicle, LPS (500 ng/mL), or LPS + Ang-(1-7) for 8 h. Then, we evaluated LC3I and LC3II protein levels through Western blot (**A**). GAPDH levels are shown as loading control. Molecular weight markers are depicted in kDa. The quantitative analysis of the experiment is shown in (**B**). The results were represented as LC3II/LC3I ratio and expressed as the mean ± S.E. (the fold of change relative to the vehicle group). (**C**) The quantitative analysis of autophagic flux was conducted using densitometric analysis of LC3II obtained by the difference between the condition with and without CQ from the representative images shown in (A). The values were normalized to the vehicle condition and expressed as the mean ± S.E. Three independent experiments were conducted (*, *p* < 0.05 vs. vehicle. #, *p* < 0.05 vs. LPS). Plus (+) and minus (−) symbols indicated in the figures or graphics mean presence or absence of treatment.

To evaluate the Ang-(1-7) effect on LPS-induced autophagy in vitro, we determined the LC3II/LC3I ratio in C2C12 myotubes incubated with LPS in the presence or absence of Ang-(1-7). We used chloroquine (CQ) to stop the autophagic flux and accumulate LC3II levels. In Figure 3A, we observed that the LC3II/LC3I ratio could be detected in the presence of CQ. Quantification of these data indicates that Ang-(1-7) at 100 nM decreased the LPS-induced increment of the LC3II/LC3I ratio (Figure 3B). We also determined the effect of Ang-(1-7) on LPS-induced autophagic flux increase, which is abolished at 100 nM of Ang-(1-7) (Figure 3C).

Additional data shows similar results of Ang-(1-7) on the LC3II/LC3I ratio (Figure S3A,B) and autophagic flux (Figure S3A,C) in undifferentiated C2C12 cells treated with LPS, showing a decrease in these parameters.

**Figure 4:**
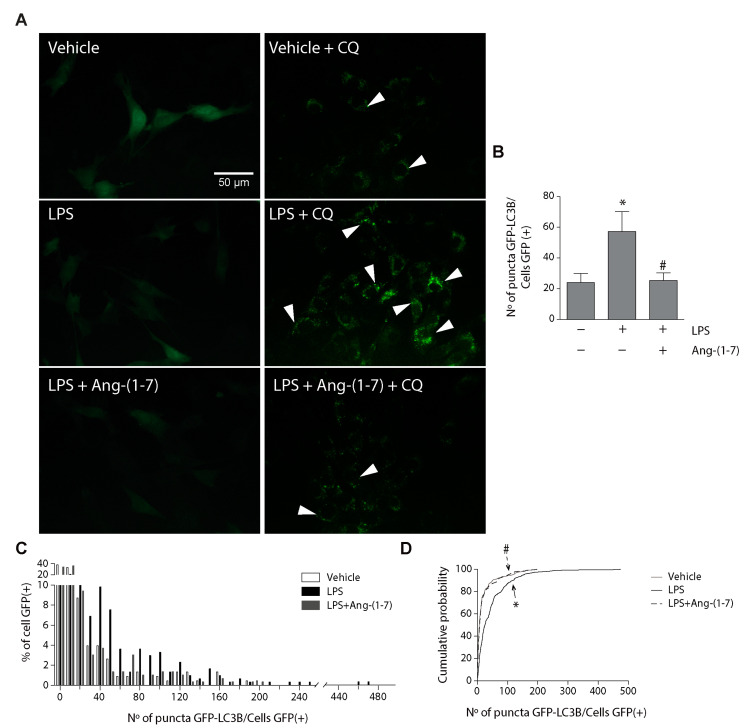
Ang-(1-7) reduced the LPS-induced LC3II/LC3I ratio and autophagic flux in C2C12 myotubes. C2C12 cells were pre-treated with or without Ang-(1-7) (1, 10, and 100 nM) for 30 min and then incubated in the absence or presence of chloroquine (CQ; 50 μM) for 5 min. Next, the cells were treated with the vehicle, LPS (500 ng/mL), or LPS + Ang-(1-7) for 8 h. Then, we evaluated LC3I and LC3II protein levels through Western blot (**A**). GAPDH levels are shown as loading control. Molecular weight markers are depicted in kDa. The quantitative analysis of the experiment is shown in (**B**). The results were represented as LC3II/LC3I ratio and expressed as the mean ± S.E. (the fold of change relative to the vehicle group). (**C**) The quantitative analysis of autophagic flux was conducted using densitometric analysis of LC3II obtained by the difference between the condition with and without CQ from the representative images shown in (**A**). The values were normalized to the vehicle condition and expressed as the mean ± S.E. (**D**) The cumulative probability distribution of autophagosomes (number of puncta) in transduced cells with Adv-GFP-LC3B incubated with CQ. Three independent experiments were conducted (*, *p* < 0.05 vs. vehicle. #, *p* < 0.05 vs. LPS). Plus (+) and minus (−) symbols indicated in the figures or graphics mean presence or absence of treatment. The white arrows are examples of autophagosomes.

We evaluated the effect of Ang-(1-7) on the LPS-induced amount of autophagosome. C2C12 cells were transduced with an adenovirus carrying a gene sequence to overexpress the fusion protein LC3B with green fluorescent protein (GFP) (Adv-LC3B-GFP), and the C2C12 cells were then treated with LPS in the absence or presence of Ang-(1-7). To demonstrate that the GFP positive puncta correspond to the autophagosome, we performed immunofluorescence to detect LC3B in cells transduced with Adv-LC3B-GFP. We observed a co-localization between signals for GFP puncta and signals for LC3B (Figure S4), which confirms that GFP signals correspond to LC3B in autophagosomes, as previously reported [15,26]. In Figure 4A, we detected autophagosomes in C2C12 cells transduced with Adv-LC3B-GFP in the absence or presence of CQ. The results of the same figure show that autophagosomes were only detected in the presence of CQ. Figure 4A shows that the basal amount of autophagosome (vehicle + CQ) increased with LPS (LPS + CQ). This increase is reduced by Ang-(1-7) (LPS + Ang-(1-7) + CQ). The quantification of autophagosome amount is shown in Figure 4B (vehicle + CQ = 24 ± 6; LPS + CQ = 57.3 ± 12.9; LPS + Ang-(1-7) + CQ = 25.3 ± 5.0). To evaluate the distribution of autophagosomes in each cell, we represented the data in a distribution frequency histogram (Figure 4C). The results show that LPS increased the percentage of cells present a higher number of autophagosomes than the control condition (vehicle). Figure 4C also shows that Ang-(1-7) prevented the effect of LPS in the increment of cells with high amounts of autophagosomes, reaching a distribution similar to that in basal conditions (vehicle). This change in frequency distribution histogram was also represented in a cumulative probability graph (Figure 4D), with a higher number of autophagosomes in cells with LPS treatment compared to LPS plus Ang-(1-7) treatment.

Together, these results indicate that Ang-(1-7) reduced the LPS-induced LC3II/LC3I ratio and autophagosomes in skeletal muscle cells.

### 2.4. Angiotensin-(1-7) Prevented the Phosphorylation of p38, JNK, and BCL-2 and the Disassembly of the Beclin1/BCL-2 Complex

**Figure 5:**
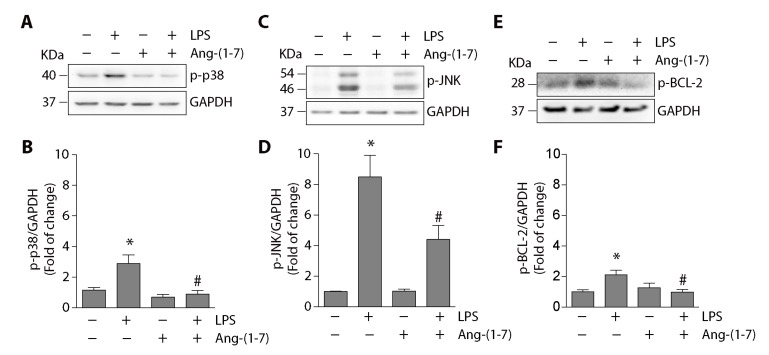
Ang-(1-7) inhibited the LPS-induced p38, JNK, and BCL-2 phosphorylation in C2C12 cells. C2C12 cells were pre-incubated in the absence or presence of Ang-(1-7) for 30 min (100 nM). Then, the cells were incubated without or with LPS (500 ng/mL) for 30 min to analyze p38 (**A**) or JNK (**C**) phosphorylation and for 4 h for BCL-2 phosphorylation (**E**) by Western blot. The levels of GAPDH are shown as loading control. Molecular weights are shown in kDa. (**B**,**D**,**F**) show the quantitative analysis of the experiments represented in (**A**,**C**,**E**), respectively. The protein levels of phospho-p38, phospho-JNK, and phospho-BCL-2 were normalized to GAPDH and expressed as the mean ± S.E. (the fold of change relative to the control). Three or four independent experiments were performed (*, *p* < 0.05 vs. vehicle. #, *p* < 0.05 vs. LPS). Plus (+) and minus (−) symbols indicated in the figures or graphics mean presence or absence of treatment.

To elucidate the intracellular mechanism involved in the reduction of LPS-induced autophagy mediated by Ang-(1-7), we analyzed the signaling pathways activated by LPS in C2C12 cells and that have been previously described to promote autophagy [11,20]. The results show that LPS increased p38 phosphorylation (2.8 ± 0.5-fold compared with the control), and Ang-(1-7) prevented this increment from reaching basal levels (0.8 ± 0.2-fold relative to the control) (Figure 5A,B). Similar results were observed for JNK phosphorylation. Figure 5C,D show that Ang-(1-7) partially prevented LPS-induced JNK phosphorylation (compared with the control: LPS = 8.4 ± 1.4-fold; LPS + Ang-(1-7) = 4.4 ± 0.9-fold).

The activation of the signaling pathway downstream p38MAPK that promotes autophagy involves the Beclin1/BCL-2 complex and specifically the BCL-2 phosphorylation in serine 70 [19]. We analyzed the effect of Ang-(1-7) on LPS-induced BCL-2 phosphorylation in C2C12 cells. The results show that LPS increased BCL-2 phosphorylation (2.1-fold compared with the control), which was reduced by Ang-(1-7) to basal levels (0.98-fold relative to the rule) (Figure 5E,F).

**Figure 6:**
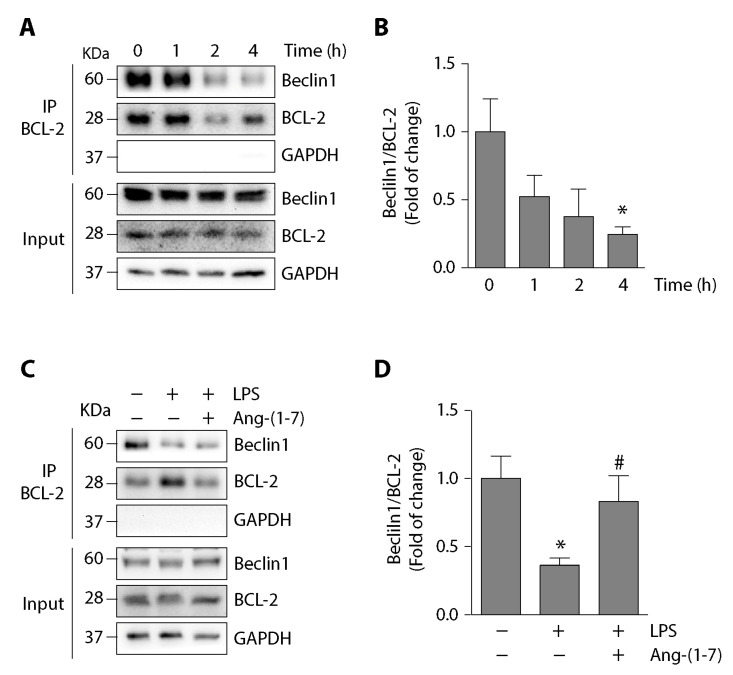
Ang-(1-7) prevented the LPS-induced disassembly of Beclin1/BCL-2 complex C2C12 cells. (**A**) C2C12 cells were incubated with LPS (500 ng/mL) at different times from 0 to 4 h. The interaction between Beclin1 and BCL-2 was analyzed using immunoprecipitation with anti-BCL-2. From the eluate, the Beclin1 and BCL-2 protein levels were detected by Western blot. The protein levels of GAPDH are shown as loading control. Molecular weights are shown in kDa. (**B**) Quantification of three independent experiments represented in (A). The Beclin1/BCL-2 ratio from the eluate was normalized to GAPDH and expressed as the mean ± S.E. (fold of change relative to time 0. *, *p* < 0.05 vs. vehicle). (**C**) C2C12 cells were pre-incubated in the absence or presence of Ang-(1-7) (100 nM) for 30 min. Then, the cells were incubated without or with LPS (500 ng/mL) for 4 h. The interaction between Beclin1 and BCL-2 was determined as in (A). (**D**) Quantification of three independent experiments represented in (C). The Beclin1/BCL-2 ratio from the eluate was normalized to GAPDH and expressed as the mean ± S.E. (the fold of change relative to the control. *, *p* < 0.05 vs. vehicle. #, *p* < 0.05 vs. LPS). Plus (+) and minus (−) symbols indicated in the figures or graphics mean presence or absence of treatment.

The MAPK-induced BCL-2 phosphorylation promotes autophagy via the disassembly of the Beclin1/BCL-2 complex [19,27]. As LPS induced BCL-2 phosphorylation, we evaluated the effect of LPS in the disassembly of the Beclin1/BCL-2 complex in skeletal muscle cells. For this, C2C12 cells were incubated with LPS at different times and evaluated by co-immunoprecipitating the Beclin1/BCL-2 interaction. The results show a reduced co-immunoprecipitation of Beclin1 with BCL-2 at 4 h after incubation with LPS (Figure 6A,B), suggesting a reduction in interaction between both proteins. Ang-(1-7) effect on the LPS-dependent reduced interaction between Beclin1 and BCL-2 was evaluated in C2C12 cells. These cells were incubated with LPS in the absence or presence of Ang-(1-7). The results show that Ang-(1-7) prevented the decreased co-immunoprecipitation of Beclin1 with BCL-2 (Figure 6C,D).

These results indicate that Ang-(1-7) reduced the LPS-induced autophagy and two downstream events: the reduction in the phosphorylation of p38, JNK, and BCL-2 and the prevention of LPS-induced disassembly of the Beclin1/BCL-2 complex.

## 3. Discussion

The RAS is a crucial regulator of skeletal muscle mass [28]. Ang-(1-7) mainly reduces Ang II-induced skeletal muscle atrophy via the Mas receptor by preventing the diminution of muscle strength, fiber diameter, and myosin heavy chain levels, which are related to an increase in UPS activation [29]. Interestingly, the Ang-(1-7)/Mas axis has also been described as an anti-atrophic peptide in Ang II-independent models of skeletal muscle atrophy, such as immobilization or sepsis induced by LPS [9,24]. In this work, we demonstrated that Ang-(1-7) could reduce autophagy via the Mas receptor in a model of LPS-induced muscle wasting. The results show that the activation of the Ang-(1-7)/Mas axis prevents the LPS-induced increase in autophagy in DFG, TA, and GA skeletal muscle. We also show that the reduction in autophagy in skeletal muscle cells is concomitant with the decrease in phosphorylation of p38, JNK, and BCL-2 and the prevention of Beclin1/BCL-2 complex disassembly, which is a pivotal event that commences autophagy.

Some studies indicate that Ang-(1-7) reduces autophagy in cells, such as fibroblasts [30], cardiomyocytes [31], and cerebrum cells [32]. In skeletal muscle, only the classical RAS components as Ang II have been demonstrated to regulate autophagy [33]. However, there are no antecedents about the regulation of autophagy by Ang-(1-7) in a model of skeletal muscle atrophy. Thus, this is the first report that shows a reduction in autophagy by Ang-(1-7) in LPS-induced skeletal muscle atrophy.

Autophagy is an essential cellular mechanism to maintain homeostasis, especially in skeletal muscle health [34,35], however, when autophagy is deregulated, it contributes to skeletal muscle atrophy through protein organelle degradation [11,12].

Sepsis, evaluated by LPS-induced animal models, has been reported to produce skeletal muscle atrophy by increasing UPS activity and p38 phosphorylation [9]. In this sense, the skeletal muscle is severely damaged by sepsis, resulting in organelles’ degradation as mitochondria through autophagy [12]. Furthermore, LPS increases autophagy with p38 phosphorylation [11]. Interestingly, one of the signaling pathways that regulate autophagy is dependent on p38, JNK, and the disassembly of the Beclin1/BCL-2 complex [36,37]. In our results, we described, for the first time, that LPS induces autophagy with an increase in p38, JNK, and BCL-2 phosphorylation and the disassembly of the Beclin1/BCL-2 complex. However, our result showing that Ang-(1-7) prevents autophagy concomitant with the abolition of p38, JNK, BCL-2 phosphorylation, and the maintenance of the Beclin1/BCL-2 complex is more critical. All these events can explain the Ang-(1-7)-dependent autophagy reduction.

Several antecedents propose p38 and JNK MAPKs as pharmacological targets that prevent skeletal muscle atrophy [9,11,38]. In this context, different reports show that the Ang-(1-7)/Mas receptor axis reduces p38 and JNK phosphorylation in the kidney and pulmonary epithelial cells [39,40], which agrees with our results. The mechanism involved in reducing MAPK phosphorylation induced by Ang-(1-7) in the skeletal muscle is unknown. One possible explanation is that Ang-(1-7) reduces p38 phosphorylation via the Mas receptor through increased activity of Src homology 2 domain-containing protein tyrosine phosphatase 1 (SHP-1), such as is reported in renal cells [39]. Another possibility is that MAP kinase phosphatase-2, activated by the Ang-(1-7)/Mas receptor axis, decreases JNK phosphorylation, as reported in pulmonary epithelial cells [40]. Another candidate is protein phosphatase 2A, activated by Ang-(1-7) [40] and reduces p38 phosphorylation. Thus, we can speculate that this phosphatase could decrease p38 phosphorylation and, subsequently, Beclin1 phosphorylation, which is responsible for preventing autophagy. Further studies must be carried out to evaluate and determine the role of phosphatases in the effect of Ang-(1-7) in autophagy and skeletal muscle atrophy.

Downstream of MAPKs, our results indicate that Ang-(1-7) reduces BCL-2 phosphorylation in serine 70. In addition to its role in autophagy, BCL-2 is described as an anti-apoptotic protein in skeletal muscle atrophy, and Ang-(1-7) prevents muscle apoptosis by preventing a decrease in BCL-2 protein levels [29]. Thus, Ang-(1-7) regulates apoptosis and autophagy by modulating BCL-2 protein levels and phosphorylation, respectively. These facts are consistent with the possibility of the two pools of BCL-2, as previously suggested [41,42].

For the first time, our results show the effect of Ang-(1-7) on the Beclin1/BCL-2 complex. Thus, our data show that Ang-(1-7) prevents the LPS-induced disassembly of the Beclin1/BCL-2 complex. We can hypothesize that BCL-2 sequesters Beclin1, avoiding the interaction of Beclin1 with PI3K class 3 to prevent PI3P and autophagosome formation, which results in a reduction in autophagy. A BH3 domain of Beclin1 mediates the interaction between Beclin1 and BCL-2. Other proteins of the BCL-2 family, such as BCL-2x_L_, Bcl-2-like protein 11, and BCL-2L1, can interact with Beclin1 and sequester it to potentially avoid autophagy [19,43]. Additionally, it has been described that AMP-activated protein kinase (AMPK) can promote the disassembly of the Beclin1/BCL-2 complex with an increase in JNK and BCL-2 phosphorylation [44]. Further experiments could be performed to evaluate the possible role of members of the BCL-2 family and AMPK in decreasing LPS-induced autophagy mediated by Ang-(1-7).

Mammalian Target of Rapamycin (mTOR) is a crucial protein amongst the different signaling pathways that promote the beginning of autophagy [45,46]. mTOR activation inhibits autophagy. Although the effect of Ang-(1-7) on mTOR activation was not evaluated in this study, antecedents suggest that mTOR could be activated by Akt, which is activated by Ang-(1-7) via the Mas receptor in skeletal muscle [24].

In our results, we have observed that p62/SQSTM1 levels are unchanged with LPS and/or Ang-(1-7) treatment. Another group has reported decreased p62/SQSTM1 protein after LPS treatment, specifically in the TA muscle, but not in the DFG and soleus [12]. The main difference between our results and this report is the higher amount of LPS injected relative to our study (20-fold higher), which could explain the possible recruitment of adaptor proteins that, under a mild treatment with LPS (as in our study), could not be participating in autophagy. Although p62/SQSTM1 has been the most studied adaptor protein in autophagy, other proteins carry out this function, such as neighbor of BRCA1 gene 1 (NBR1) and optineurin (OPTN) [47,48]. Thus, p62-independent autophagy has been reported in inflammatory diseases, such as sepsis [48,49]. Studies on skeletal muscle have reported the role of NBR1 and OPTN in autophagy [50,51,52]. Therefore, further research could be performed to analyze and identify other adaptor proteins that participate in LPS-induced autophagy in skeletal muscle.

An important observation from our results is based in the relative levels of autophagic markers which suggest that there is a higher autophagy in the Mas KO mice than in WT mice. As Ang-(1-7) can decrease autophagy, one possible explanation for our results is that in WT mice, the basal levels of Ang-(1-7) could be acting on muscle autophagy. In Mas KO mice, this effect of Ang-(1-7) on autophagy is lost and can produce a higher basal or LPS-induced autophagy than in WT mice.

## 4. Materials and Methods

### 4.1. Animals and Experimental Protocols

Twelve-week-old male C57BL/6J wild-type (WT) and Mas knockout (Mas KO) were used [53]. The animals were randomized and separated into the following experimental groups for WT, or Mas KO mice (9–12 animals/group) were designed according to their treatments: PBS (Vehicle), LPS, Ang-(1-7), LPS plus Ang-(1-7) (LPS + Ang-(1-7)). A single sub-clinical dose of LPS (1 mg/kg) was intraperitoneally (i.p.) injected [9,11]. The treatments were performed by 18 h, except for muscle strength (14 days) or contractile measurements (24 h) such as we and others have previously reported [9,54]. The Ang-(1-7) peptide (100 ng/kg/min) was administrated through osmotic micropumps (Alzet-Durect, Cupertino, CA, USA) accordingly to our previous reports [9]. At the end of the experiment, the animals were euthanized under anesthesia, and the diaphragm (DFG), tibialis anterior (TA), and gastrocnemius (GA) muscles were dissected, removed and rapidly frozen, and stored at −80 °C until processing. All the experiments and protocols were carried out as per the Animal Research: Reporting of In Vivo Experiments (ARRIVE) guidelines and following the National Institutes of Health (NIH) guide for the care and use of Laboratory animals (revised 1978). Under the Animal Ethics Committee’s formal approval at the Universidad Andrés Bello (Approval number 030/2012, date: 20 August 2012).

### 4.2. Strength Test

At the end of treatment, mice were analyzed to measure forelimb force with a weightlifting test described previously [55]. The mice must briefly maintain with its forepaws chain links with increasing weights attached to a ball of tangled fine wire. They were seven different weights from 15 to 55 g. Previous to the test, the mice were trained once per day for two weeks. The mouse grasps the different weights with forepaws to perform the analysis, and the score is assigned. The final score was calculated as the summation of the product between the link weight and the time held. The average of three measures from each mouse was normalized by body weight. All the steps were performed blindly.

### 4.3. Contractile Measurements

After treatment, the mice were anesthetized, the DFG muscles were removed, and the muscle contractile properties were measured as previously described [56,57]. The maximum isometric tetanic force was determined. The muscle mass and the optimum muscle length (Lo) were used to calculate the specific net force (force normalized per total muscle fiber cross-sectional area [CSA], mN/mm^2^). All the contractile measures were performed blindly.

### 4.4. Cell Cultures

The skeletal muscle cell line C2C12 (American Type Culture Collection, Manassas, VA, USA) was grown and differentiated until day 4, as described previously [58]. The myotubes were pre-incubated with Ang-(1-7) (1 up to 100 nM) (Sigma-Aldrich, St Louis, MO, USA) at 30 min before the incubation with chloroquine (CQ) (50 μM) (Sigma-Aldrich, St Louis, MO, USA) for 5 min before LPS treatment for 8 h. Both Ang-(1-7) and CQ were maintained during the treatment with LPS [500 ng/mL from *Escherichia coli* 0127:B8 (Sigma-Aldrich, St Louis, MO, USA)]. The evaluation of intracellular signaling pathways was performed in C2C12 cells, which were pre-incubated with Ang-(1-7) (100 nM) for 30 min before LPS treatment (500 ng/mL). Ang-(1-7) was maintained during incubation with LPS.

### 4.5. Immunoblot Analysis

DFG, TA, and GA muscles were homogenized in Tris-ethylenediaminetetraacetic acid (EDTA) buffer (50 mM Tris, 10 mM EDTA, pH 8,3) with a cocktail of protease inhibitors (Sigma-Aldrich, St Louis, MO, USA) and 1 mM phenylmethylsulfonyl fluoride (PMSF) (Sigma-Aldrich, St Louis, MO, USA). C2C12 cells were homogenized in radioimmunoprecipitation assay (RIPA) buffer with a cocktail of protease inhibitors and 1 mM PMSF. Proteins were separated into SDS-PAGE, transferred onto polyvinylidene difluoride (PDVF) membranes (Merck, Temecula, CA, USA), and probed with rabbit anti-LC3B (1:1000, Cell Signaling, Danvers, MA, USA), rabbit anti-phospho-p38 (1:1000, Cell Signaling, Danvers, MA, USA), rabbit anti-phospho-JNK (1:1000, Cell Signaling, Danvers, MA, USA), rabbit anti-phospho-BCL-2 (1:1000, Cell Signaling, Danvers, MA, USA), rabbit anti-Beclin1 (1:1000, Cell Signaling, Danvers, MA, USA), rabbit anti-BCL-2 (1:500; Santa Cruz, Dallas, TX, USA) and mouse anti-GAPDH (1:2000; Santa Cruz, Dallas, TX, USA). All immunoreactions were visualized by enhanced chemiluminescence (Thermo Scientific, Waltham, MA, USA) which were detected through an image documentation system, Fotodyne (Fisher Scientific, St. Waltham, MA, USA).

### 4.6. Reverse Transcription and Quantitative Real-Time PCR

Total RNA was isolated from the DFG muscle using TRIzol (Thermo Scientific, Waltham, MA, USA). The total RNA (1 μg) was reverse transcribed to cDNA using random hexamers and Superscript II reverse transcriptase (Thermo Scientific, Waltham, MA, USA). TaqMan quantitative real-time PCR was performed in triplicate, using an Eco Real-Time PCR System (Illumina, San Diego, CA, USA) with pre-designed primers for mouse *lc3b*, *ctsl* and the housekeeping gene *β-actin* (TaqMan Assays on-Demand; Thermo Scientific, Waltham, MA, USA). The mRNA expression was quantified using the comparative ∆CT method (2-∆∆CT), with β-actin as the reference gene [59]. The mRNA levels were expressed relative to the mean expression in the vehicle-treated mice [60].

### 4.7. Co-Immunoprecipitation Analysis

C2C12 cells were homogenized in co-immunoprecipitation buffer (Tris 20 mM, NaCl 137 mM, EDTA 2 mM, NP-40 1%), with a cocktail of protease inhibitors (Sigma-Aldrich, St Louis, MO, USA) and 1 mM PMSF (Sigma-Aldrich, St Louis, MO, USA). Immunoprecipitation of endogenous BCL-2 was performed overnight at 4 °C using rabbit anti-BCL-2. The immune complex was precipitated with protein an agarose plus beads (Thermo Scientific, Waltham, MA, USA) for 1 h at 4 °C. SDS-PAGE separated eluates from precipitated beds, and Beclin1, BCL-2, or GAPDH were detected by Western blot.

### 4.8. Indirect Immunofluorescence

C2C12 cells were grown, transduced with adenovirus, and treated on glass coverslips. At the end of the experiment, cells were washed twice in ice-cold PBS-Ca^+2^/Mg^+2^, fixed in methanol, blocked for 1 h in 10% (*v*/*v*) goat serum in PBS, and incubated for 1 h with specific antibody rabbit anti-LC3B (1:200; Cell Signaling, Danvers, MA, USA). The bound antibodies were detected by incubating the cells for 1 h with 1:10,000 affinity-purified Alexa Fluor dye-conjugated goat anti-rabbit antibody (Thermo Scientific, Waltham, MA, USA). After rinsing, the cells were mounted with fluorescence mounting medium (Agilent Dako, Santa Clara, CA, USA) under a glass slide and viewed and photographed using the Motic BA310 fluorescence microscopy (Motic, Hong Kong, China).

### 4.9. Adenovirus Transduction and Analysis of GFP-LC3B Puncta

C2C12 cells were grown and transduced with an adenovirus carrying the sequence for expression of GFP-LC3B (Adv-GFP-LC3) [61]. Briefly, cells were transduced at a multiplicity of infection (MOI) of 1000 and maintained for 16 h. After that, the medium was removed, and a fresh medium supplemented with 10% *v/v* of fetal bovine serum was added. After 20 h, incubation with LPS, CQ, and/or Ang-(1-7) was performed as was described in each figure. Images were acquired with Motic BA310 fluorescence microscopy (Motic, Hong Kong, China). The number of GFP-LC3B puncta was determined by using customized ImageJ software (NIH, Bethesda, MD, USA). Analysis of these data was performed to determine the mean of autophagosome per cells, percentage of distribution, and cumulative probability of cells with autophagosome.

### 4.10. Autophagy Assay

The protein levels of LC3I, LC3II, and p62 were detected by Western blot. The LC3II/LC3I ratio was analyzed as a parameter of autophagy. Autophagic flux was determined by analysis of LC3II protein levels in cells incubated with chloroquine (CQ), as described previously [16]. The amount of autophagosome was determined in C2C12 cells expressing GFP-LC3B by transduction with Adv-GFP-LC3B [15]. The fluorescent detection of GFP was performed using the BA310 fluorescence microscopy (Motic, Hong Kong, China).

### 4.11. Statistics

For statistical analysis, we used a t-test to compare two groups. To compare three or more groups, we used one or two-way analysis of variance (ANOVA) with a post hoc Bonferroni’s multiple-comparison test (Prism 8, GraphPad Software, San Diego, CA, USA). To analyze the accumulative frequency of fiber sizes, we used Kruskal-Wallis and Wilcoxon test (IBM SPSS Statistics Software. IBM Corp., New York, NY, USA). A difference was considered statistically significant at *p* value < 0.05, as is indicated in each figure.

## 5. Conclusions

In conclusion, this study reports, for the first time, a reduction in autophagy by Ang-(1-7) via the Mas receptor in an LPS-induced model of skeletal muscle wasting. We also demonstrate in C2C12 culture cells that Ang-(1-7) prevents LPS-induced key events that could be related to the increase of autophagy, namely, the activation of signaling pathways, such as p38 and JNK MAPKs, and the formation of the Beclin1/BCL-2 complex.

## Supplementary Materials

Supplementary Materials can be found at https://www.mdpi.com/1422-0067/21/24/9344/s1.

## Abbreviations

AdV-GFP-LC3	Adenovirus for GFP-LC3
AMPK	AMP-activated protein kinase
Ang-(1-7)	Angiotensin (1-7)
Atrogin-1	atrogin-1/MAFbx
AT1	Angiotensin II receptor type 1
BCL-2	B-cell lymphoma-2
CQ	Chloroquine
DFG	Diaphragm
EDTA	Ethylenediaminetetraacetic acid
GA	Gastrocnemius
GAPDH	Glyceraldehyde-3-phosphate dehydrogenase
GFP	Green fluorescent protein
JNK	c-Jun N-terminal kinase
LC3B	Microtubule-associated proteins 1A/1B light chain 3B
LPS	Lipopolysaccharide
kDa	Kilodalton
KO	Knock out
MAPK	Mitogen-activated protein kinase
Mas KO	Mas knockout (deficient in Mas receptor)
MOI	Multiplicity of infection
MuRF-1	Muscle RING-finger protein-1
NBR1	Neighbor of BRCA1 gene 1
OPTN	Optineurin
PE	Phosphatidylethanolamine
p62/SQSTM1	Sequestosome 1
PI3P	Phosphatidylinositol 3-phosphate
PMSF	Phenylmethylsulfonyl fluoride
PVDF	Polyvinylidene fluoride
qPCR	Quantitative polymerase chain reaction
RAS	Renin-angiotensin system
RIPA	Radioimmunoprecipitation assay
ROI	Region of interest
ROS	Reactive oxygen species
RT	Reverse transcription
RAS	Renin angiotensin system
SHP-1	Src homology 2 domain-containing protein tyrosine phosphatase 1
TA	Tibialis anterior
UPS	Ubiquitin proteasome system
WT	Wild-type
