# Supplementary material for: Angiotensin-(1-7) Prevents Lipopolysaccharide-Induced Autophagy via the Mas Receptor in Skeletal Muscle
Source: Int J Mol Sci. 2020 Dec 8;21(24):9344. doi: 10.3390/ijms21249344 (PMC7762589; doi:10.3390/ijms21249344)
Supplement: Supplementary file 1 [file ijms-21-09344-s001.pdf]

International Journal of *Molecular Sciences*

MDPI

Article

Angiotensin-(1-7) Prevents Lipopolysaccharide-Induced Autophagy via the Mas Receptor in Skeletal Muscle

Juan Carlos Rivera [1,2,3], Johanna Abrigo [1,2,3], Franco Tacchi [1,2,3], Felipe Simon [2,4,5], Enrique Brandan [6,7], Robson A. Santos [8], Michael Bader [9,10,11,12], Mario Chiong [13], and Claudio Cabello-Verrugio [1,2,3,*]

[1] Laboratory of Muscle Pathology, Fragility and Aging, Department of Biological Sciences, Faculty of Life Sciences, Universidad Andres Bello, Santiago 8370146, Chile; jcfriverac@gmail.com (J.C.R.); j.abrigo.leon@gmail.com (J.A.); f.tacchifernandez@uandresbello.edu (F.T.)

[2] Millennium Institute on Immunology and Immunotherapy, Santiago 8370146, Chile; fsimon@unab.cl

[3] Center for the Development of Nanoscience and Nanotechnology (CEDENNA), Universidad de Santiago de Chile, Santiago 8350709, Chile

[4] Laboratory of Integrative Physiopathology, Department of Biological Sciences, Faculty of Life Sciences, Universidad Andres Bello, Santiago 8370186, Chile

[5] Millennium Nucleus of Ion Channel-Associated Diseases (MiNICAD), Universidad de Chile, Santiago 8380453, Chile

[6] Center for Cell Regulation and Pathology (CRCP), Center for Regeneration and Aging (CARE), Laboratory of Cell Differentiation and Pathology, Department of Cell and Molecular Biology, Faculty of Biological Sciences, P. Universidad Católica de Chile, Santiago 8330077, Chile; ebrandan@bio.puc.cl

[7] Fundación Ciencia & Vida, Santiago 7780272, Chile.

[8] National Institute in Science and Technology in Nanobiopharmaceutics, Department of Physiology and Biophysics, Federal University of Minas Gerais (UFMG), Belo Horizonte 31270-901 Brazil; robsonsant@gmail.com

[9] Max-Delbrück-Center for Molecular Medicine, Berlin-Buch 13125, Germany; mbader@mdc-berlin.de

[10] Institute for Biology, University of Lübeck, Lübeck 23562, Germany

[11] Charité University Medicine, Berlin 10117, Germany

[12] German Center for Cardiovascular Research (DZHK), Berlin 10785, Germany

[13] Advanced Center for Chronic DiSeases (ACCDiS), Universidad de Chile, Santiago 8380492, Chile; mchiong@ciq.uchile.cl

* Correspondence: claudio.cabello@unab.cl; Tel.: +5622-770-3665

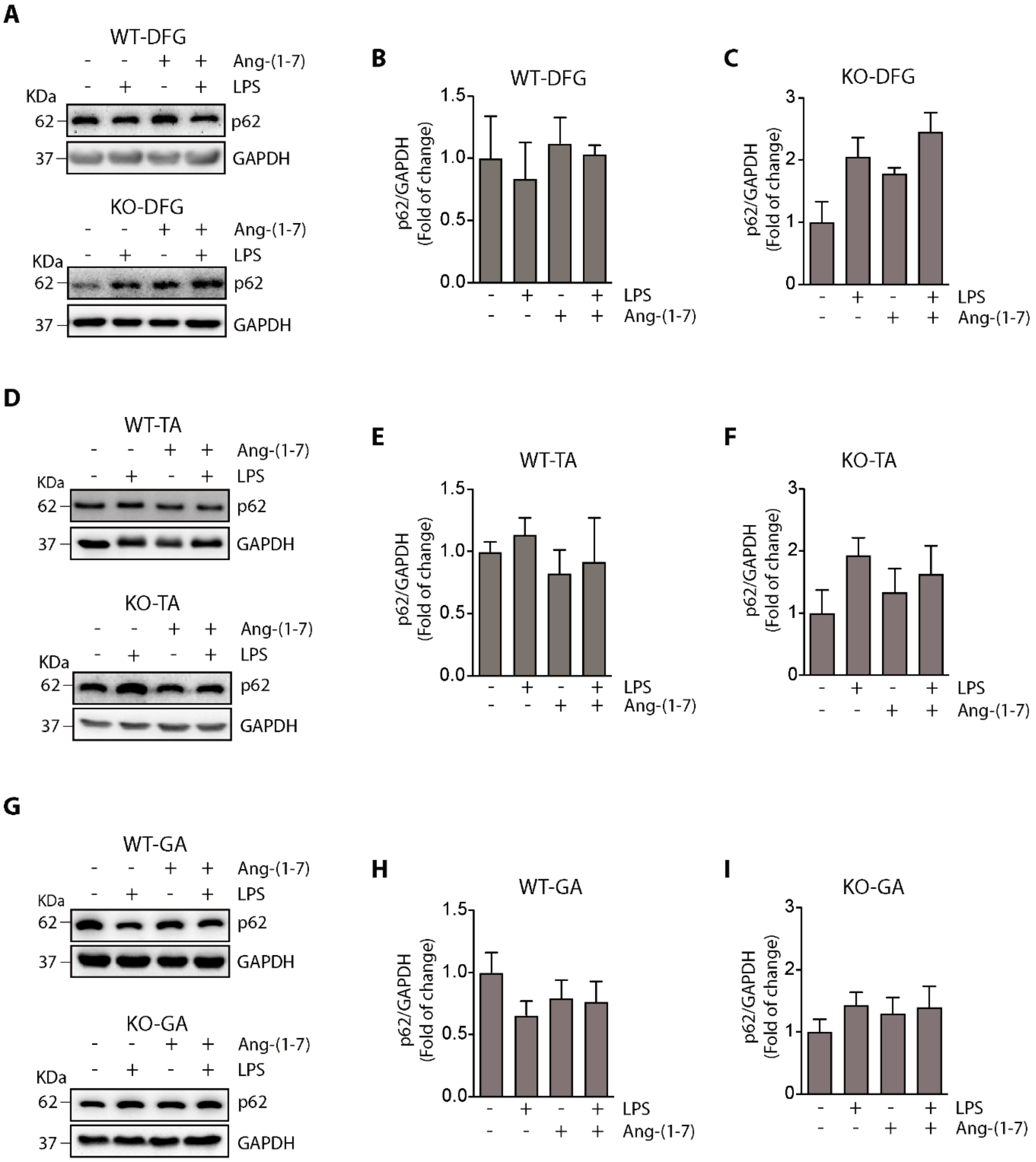

Figure S1. Treatment with LPS and/or Ang-(1-7) does not change the p62/SQSTM1 protein levels in muscles from mice. C57BL/6J wild-type or Mas KO male mice were treated with the vehicle, LPS, Ang-(1-7) or LPS+Ang-(1-7) for 18 h. When the experiments were finished, the mice were sacrificed, and the muscle was excised and homogenised to evaluate p62/SQSTM1 protein levels through Western blot analysis in DFG **(A)**, TA **(D)** and GA **(G)**. GAPDH levels are shown as the loading control (The images for GAPDH are the same as those for Figure 2). Molecular weight markers are depicted in kDa. The quantitative analysis of the experiments is shown for DFG **(B, C)**, TA **(E, F)** and GA **(H, I)**. The results were expressed as the mean ± S.E. (the fold of change relative to the vehicle group). Nine animals per group were used for these experiments (*, $P < 0.05$ vs. vehicle. #, $P < 0.05$ vs. LPS).

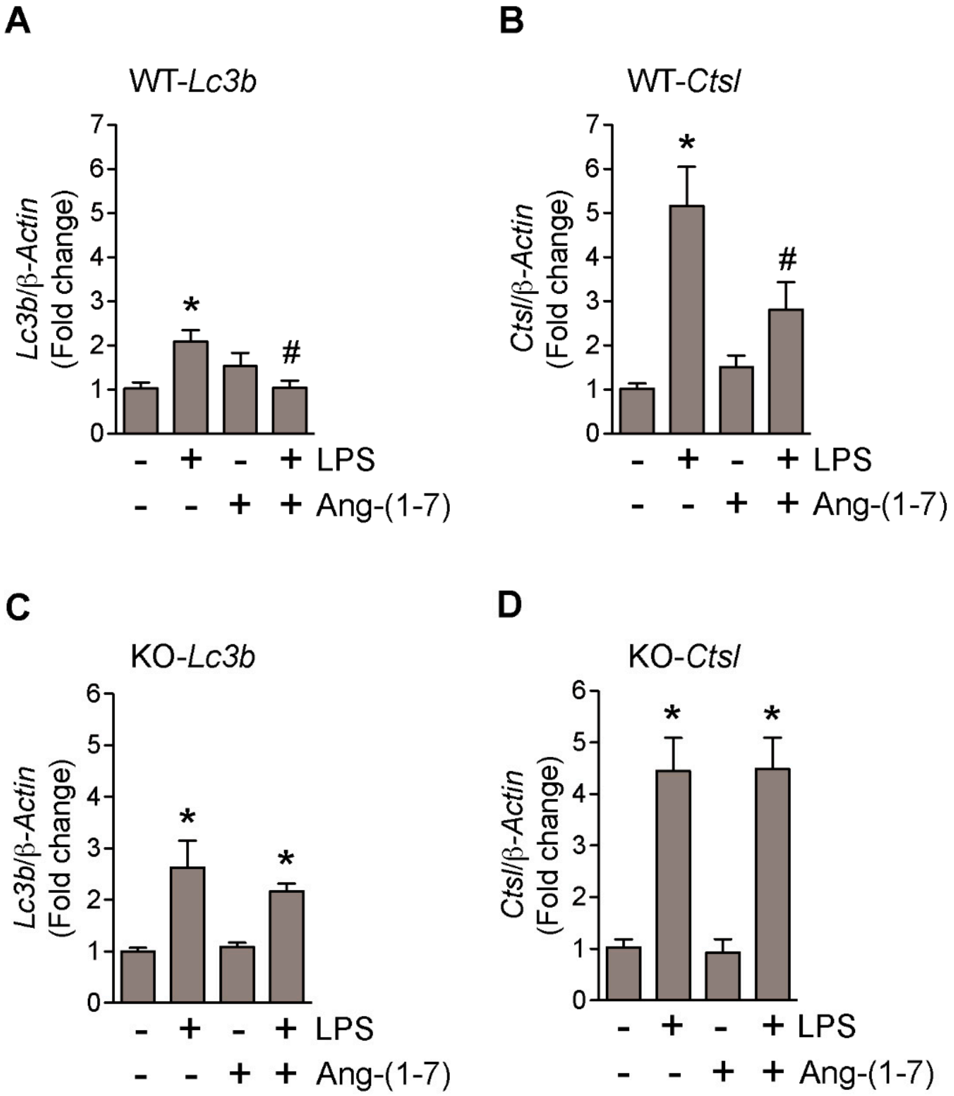

Figure S2. Ang-(1-7) reduced LPS-induced *Lc3b* and *Ctsl* gene expressions via the Mas receptor in muscles from mice. *Lc3b* and *Ctsl* gene expressions were evaluated by RT-qPCR in DFG from WT **(A, B)** and KO Mas **(C, D)** mice treated with the vehicle, LPS, Ang-(1-7) or LPS+Ang-(1-7) for 18 h. The graph shows the mRNA levels of *Lc3b* **(A, C)** and *Ctsl* **(B, D)** normalised to the β-actin levels used as the loading control. The levels are represented as the mean ± S.E. (the fold of change relative to the vehicle). Nine to twelve animals per group were used in these experiments (*, P < 0.05 vs. vehicle; #, P < 0.05 vs. LPS).

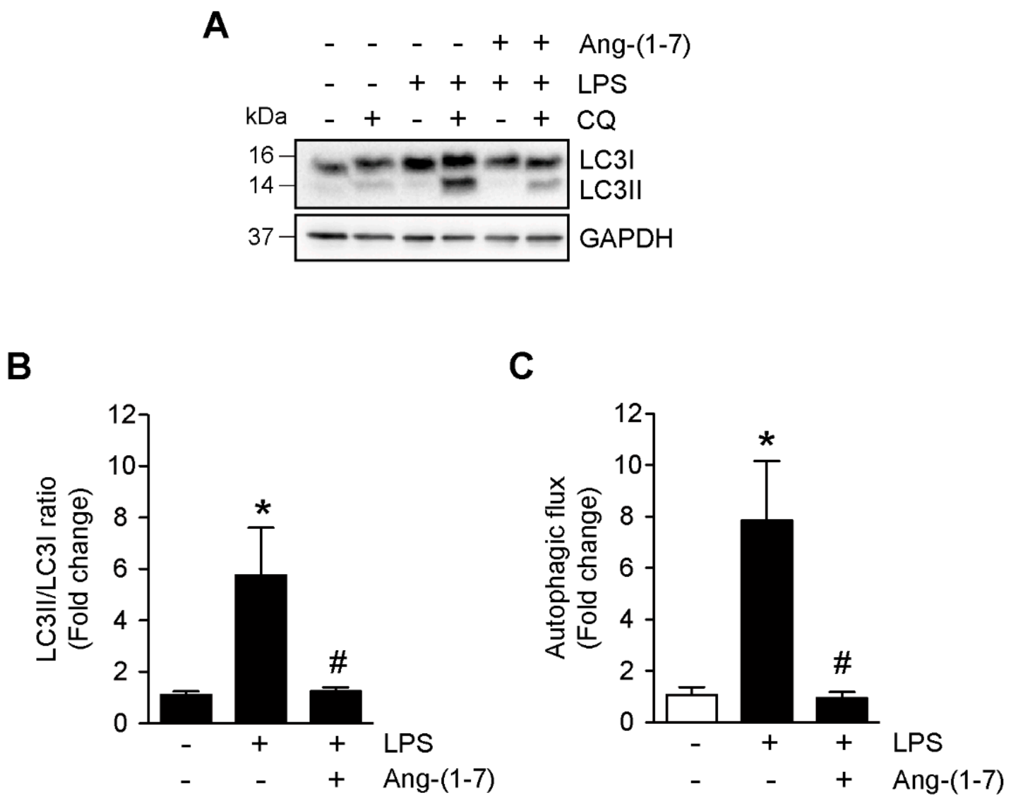

Figure S3. Ang-(1-7) reduced LPS-induced autophagy in C2C12 myoblasts. C2C12 cells were pre-incubated without or with of Ang-(1-7) (100 nM) for 30 min. Then, the cells were incubated with or without CQ (50 µM) for 5 min. Finally, the cells were incubated without or with LPS (500 ng/mL) for 8 h. **(A)** LC3I and LC3II protein levels were detected by Western blot analysis. The levels of GAPDH are shown as the loading control. Molecular weights are shown in kDa. **(B)** Quantitative analysis of three independent experiments represented in **(A)** of treatments with CQ. The results were described as the LC3II/LC3I ratio and expressed as the mean ± S.E. (the fold of change relative to the control group. *, P < 0.05 vs. vehicle. #, P < 0.05 vs. LPS). **(C)** Autophagic flux derived from **(A)**. The value of LC3II was normalised to GAPDH and expressed as the mean ± S.E. (the fold of change relative to the control group. *, P < 0.05 vs. vehicle. #, P < 0.05 vs. LPS).

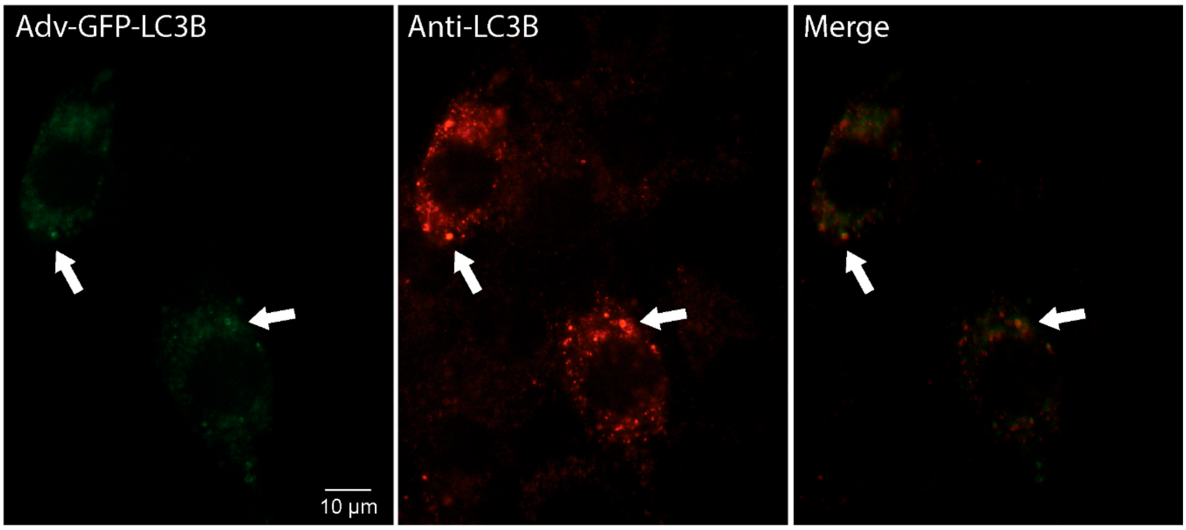

Figure S4. Colocalization of overexpressed GFP-LC3B with endogenous LC3B in LPS-induced autophagosomes *in vitro*. C2C12 cells were transduced with Adv-GFP-LC3. The cells were incubated with CQ (50 µM) for 5 min and further incubated with LPS (500 ng/mL) for 8 h. At the end of the experiment, fluorescent detection of GFP (left) and LC3B was performed using indirect

immunofluorescence with anti-LC3B (centre). The colocalisation of overexpressed GFP with endogenous LC3B is shown (right). The arrows indicate examples of autophagosomes (puncta).
